# AO Spine Clinical Practice Recommendations: Sagittal Alignment Assessment and Planning in Adult Spinal Deformity Surgery

**DOI:** 10.1177/21925682261452195

**Published:** 2026-05-27

**Authors:** Anna Rienmueller, Colby Oitment, Thorsten Jentzsch, Alisson Teles, Jeffrey Hills, Charles Fisher, Caglar Yilgor, Michael Kelly, AO Spine Knowledge Forum Deformity

**Affiliations:** 1 Department of Orthopedic Surgery, Medical University Vienna, Vienna, Austria; 2 Scarborough General Hospital (SGH), Scarborough Health Network, 12366 University of Toronto, Faculty of Medicine, Toronto, ON, Canada; 3 University Spine Center Zurich, Department of Orthopedics, 31031 Balgrist University Hospital, University of Zurich, Zurich, Switzerland; 4 124919 Santa Casa de Porto Alegre, Porto Algere, Brazil; 5 14742 University of Texas Health San Antonio, San Antonio, TX, USA; 6 Division of Spine Surgery, University of British Columbia and Vancouver General Hospital, Vancouver, BC, Canada; 7 587267 Koc University Hospital Spine Center, Istanbul, Türkiye; 8 14444 University California San Diego and Rady Children’s Hospital, San Diego, CA, USA; 9 AO Innovation Translation Center, AO Foundation, Davos, Switzerland

**Keywords:** adult spine deformity, sagittal plane alignment, Roussouly, SRS Schwab, GAP score, T4–L1–Hip axis, literature review

## Abstract

**Study Design:**

Literature review with clinical recommendation.

**Objectives:**

To provide the readers with a concise curation of the relevant spine literature regarding patient-specific alignment planning in patients with adult spinal deformity (ASD) and set out recommendations for how the practicing clinician should interpret and make use of this evidence.

**Methods:**

Key articles on patient-specific alignment planning for ASD were reviewed to develop clinical recommendations by consensus. Recommendations are graded as strong or conditional, based on methodological quality and expert opinion.

**Results:**

Four articles were selected by the AO Spine Knowledge Forum Deformity and each evaluated for the strength of methodology and scientific evidence.

**Conclusions:**

The 4 reviewed publications illustrate the progression from descriptive to proportional and finally continuous alignment concepts in adult spinal deformity surgery. The Roussouly morphotypes help clinicians understand native sagittal shape and compensatory patterns, the SRS–Schwab classification remains useful for standardized description and communication, the GAP Score introduces pelvic-incidence–based proportionality, the T4–L1–Hip axis offers continuous, directly modifiable angular targets. Used together, these models offer complementary perspectives that enhance preoperative planning and postoperative evaluation. Integrating their strengths, while considering patient-specific factors such as bone quality, physiologic reserve, and surgical goals, supports more individualized and durable alignment strategies.

## Introduction

Adult spinal deformity (ASD) correction aims to restore spinal alignment to improve function, reduce disability, and minimize mechanical complications—while respecting patient-specific variables such as comorbid conditions and their ability to tolerate an ASD reconstruction. A prevailing principle is the need for improvement, and possibly normalization, in the sagittal plane as sagittal malalignment, even slight, increases energy expenditure and risk of decompensation.^1,2^ Sagittal balance is, however, not a static geometric goal but rather a dynamic biomechanical equilibrium.^3^ A prevailing challenge in ASD surgery is not whether alignment matters, but rather how to define the right alignment for a given patient.

Historically, alignment strategies have relied on radiographic targets such as the C7 sagittal vertical axis (SVA), pelvic tilt (PT), and pelvic incidence-lumbar lordosis (PI–LL) mismatch—as codified in the SRS–Schwab classification.^4^ Although these parameters offer simplicity, postural measures such as PT and SVA cannot be measured intraoperatively, and a normal PI-LL mismatch varies with PI. Subsequent alignment models have introduced more nuanced, pelvic incidence-driven parameters, such as the Global Alignment and Proportion (GAP) score^5^ and the T4–L1–Hip axis,^6^ aiming to individualize target alignment which is a first step towards a precision medicine approach to ASD care.

Restoring normal alignment does not guarantee perfect outcomes. A comprehensive understanding of individual alignment, physiologic tolerance, and tissue properties is essential to minimize, and not eliminate, the risk of complications such as proximal junctional kyphosis (PJK) and implant failure.^7,8^

This review synthesizes 4 key publications representing the evolution of sagittal alignment understanding and contextualizes their utility, limitations, and applicability in current practice. We present these papers so that the reader can understand the history of sagittal plane deformity concepts, which have evolved from descriptive categories and “types” to individualized categories of alignment based upon pelvic incidence, and finally continuous measures of alignment in the pursuit of a precision-medicine approach to the sagittal plane. Based on this evidence and expert consensus, we offer conditional recommendations that integrate current tools with individualized judgment, while outlining areas requiring future refinement. (Table 1)

**Table 1.** Comparison of Alignment Models With Recommendation

Model	Main concept	Strengths	Limitations	Clinical application	Recommendation	Grade
Roussouly Morphotypes^8^	Describes natural variability in sagittal alignment based on sacral slope and lumbar apex.	• Morphological foundation, • Useful for anticipating compensation.	• Not directly surgical, • No target parameters.	Understanding of native shape and spinopelvic harmony.	**Conditional:** Integrate morphotype understanding into surgical strategy to maintain spinopelvic harmony.	Moderate
SRS–Schwab Classification^3^	Descriptive radiographic classification using PI–LL, PT, and SVA.	• Simple, • Reproducible, • Widely adopted.	• No predictive value, • Lacks individualization, • Uses postural measures, • Uses classification.	Baseline deformity severity description.	**Conditional:** Use as baseline classification for documentation and description of radiographic deformity.	Low
GAP Score^4^	Described spinopelvic alignment with pelvic-incidence specific measures.	• Introduces patient morphology into planning, • Risk stratification.	• External validity challenged, • Uses postural measures, • Uses classification.	Tool that allows precise analysis of spinopelvic alignment.	**Conditional:** Use for planning spinopelvic alignment, after surgery it can be applied to estimate risk of mechanical failure; use with caution due to limited external validation.	Moderate
T4–L1–Hip Axis^5^	Provides thoracolumbar alignment targets using PI, L1PA and T4PA, based on risk for mechanical complication.	• Based on normal alignment, • Used nonlinear probability model with metrics that can be measured intraoperatively.	• Less validated, • Not comprehensive for full spinal alignment, • Only applies to long fusions from upper thoracic spine to the sacrum.	Instrumented fusions from upper thoracic spine to the sacrum/pelvis.	**Conditional:** Apply to guide thoracic and lumbar regional alignment, adjusting for PI, to minimize risk of mechanical complications.	Moderate

## Methods

The AO Spine Knowledge Forum Deformity selected articles based on their relevance to surgical alignment planning in ASD. Each was critically appraised using a standardized format:

• Clinical Rationale
• Study Summary
• Methodological Review
• Quality of Evidence (High, Moderate, Low, Very low)
• Strength of Recommendation (Strong, Conditional)

Consensus was reached in a AO Spine Knowledge Forum Deformity meeting held July 30, 2025. Methodological limitations and clinical gaps were explicitly considered when grading recommendations. The evidence was graded according to the GRADE Handbook 2013, and recommendation was developed based on strength of evidence and expert opinion individually for each article.^9^

**Article 1– Roussouly P, Gollogly S, Berth** **onnaud E, Dimnet J. Classification of the Normal Variation in the Sagittal Alignment of the Human Lumbar Spine and Pelvis in the Standing Position. Spine. 2005;30(3):346-353.^10^**

### Clinical Rationale

**Figure 1.**
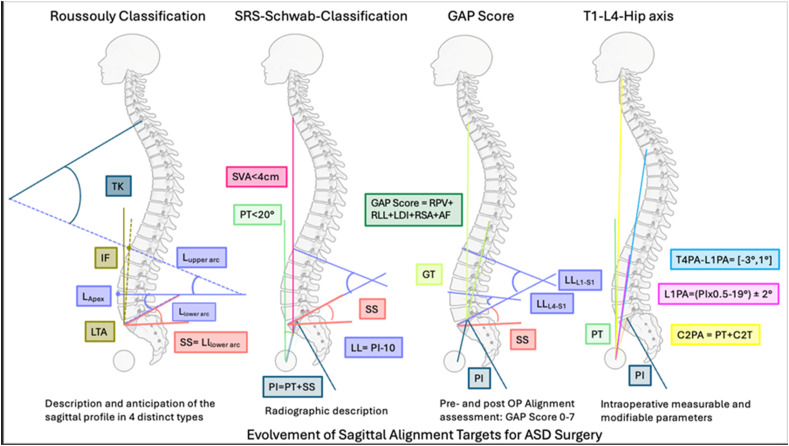
Evolvement of Sagittal Alignment Targets for ASD Surgery: TK = thoracic kyphosis, LL = Lumbar Lordosis, SS = Sacral Slope, IF = Inflection point, PI = Pelvic Incidence, PT = Pelvic Tilt, LTA = Lumbar Tilt Angle, L_Apex_ = Apex of lumbar Lordosis, GT = Global Tilt, AF = Age Factor, RPV = Relative Pelvic Version = SS-(PI x 0.59 + 9), RLL = Relative Lumbar Lordosis = LL – (PI x 0.62 + 29), LDI = (LL_L4-S1_/LL_L1-S1_) x 100, RSA = Relative Sagittal Angle = GT-(PI x 0.48-15)

Sagittal alignment in asymptomatic adults varies widely, and that variability may influence degenerative changes and subsequent sagittal alignment patterns. This study aimed to develop a classification system for sagittal spinal alignment in asymptomatic adults by considering sacral slope. Understanding the spectrum of Roussouly types may influence surgical strategies and interpretation of spinal pathologies (Figure 1).

### Study Summary

The study was a prospective radiographic analysis of 160 asymptomatic adult volunteers. Full-length anteroposterior and lateral films were obtained; a custom software tool generated geometric measures of spinal and pelvic alignment and grouped subjects into 4 sagittal morphotypes (Type 1-4) based on sacral slope. These types are strongly related to particular lordotic and kyphotic arcs, with corresponding locations of both apices and inflection points.

### Methodological Review

Strengths of this study include prospective enrollment, standardized positioning, defined inclusion/exclusion criteria (asymptomatic, no major spinal pathology), and systematic digital measurement of sagittal and pelvic parameters. Limitations include selection bias with a young, all-European volunteer cohort (which limits external validity), and the absence of a relationship with surgical outcomes. The authors note the need for longitudinal follow-up to link morphotype with symptoms or degeneration.

### Quality of Evidence: Moderate

#### Recommendation for Integrating Into Clinical Practice

The Roussouly classification is a practical method for describing sagittal morphology and lumbopelvic relationships during assessment and surgical planning. Recognizing a patient’s baseline type may help set alignment targets and anticipate compensatory patterns to optimize results. It is important to recognize that preoperative Roussouly type is less important than understanding the target shape/postoperative Roussouly type.

### GRADE Recommendation: Conditional

**We suggest** using the Roussouly classification as a practical tool to describe and anticipate sagittal profiles. Subsequent papers describe the degeneration patterns associated with particular types and may inform alignment targets to restore an appropriate shape. However, the classification tends towards poor inter-rater reliability, complicating broad adoption.

**Article 2 – Schwab, F., et al., Scoliosis Research Society-Schwab adult spinal deformity classification: a validation study. Spine (Phila Pa 1976), 2012. 37(12): p. 1077-82^4^**

### Clinical Rationale

The study aimed to develop a reliable, reproducible, and radiographically based classification for ASD. By including coronal curve types and pelvic parameters (pelvic incidence (PI)-lumbar lordosis (LL), sagittal vertical axis (SVA), and pelvic tilt (PT)), the system aimed to enhance inter-rater reliability in describing deformities. This system aimed to standardize ASD evaluation and communication by integrating pelvic parameters that correlate with disability. It offered clinicians a simplified framework to describe deformity severity (Figure 1).

### Study Summary

An ASD classification system was developed and tested using 21 cases rated by 9 experienced surgeons. Curves were classified by coronal curve type, then modifiers described pelvic tilt (PT), PI–LL mismatch, and sagittal vertical axis (SVA). Each modifier was divided into 1 of 3 groups. Inter- and intra-rater agreement averages were excellent for all modifiers (κ > 0.80), supporting reproducibility.

### Methodological Review

Strengths of this study include a structured radiographic framework, strong reliability statistics, and immediate clinical utility. However, it classifies ASD in descriptive categories for communication but does not inform surgical decision making. Despite subsequent adoption of the modifiers as alignment targets, this paper did not offer such targets. Finally, despite emphasizing the relationship between PI and lumbar alignment, the system does not consider the variable relationship between PI and the categories created (eg, PI-LL mismatch, PT). As a result, the SRS–Schwab classification remains most useful as a standardized descriptive framework rather than a tool for patient-specific alignment planning or risk stratification.

### Quality of Evidence: Low

#### Recommendation for Integrating Into Clinical Practice

##### GRADE Recommendation: Conditional

**We suggest** using the SRS-Schwab classification as a radiographic description of ASD phenotypes. The system is limited as our understanding of the relationship between PI and the various modifiers has improved. It should not be used for alignment planning, as it does not account for compensatory mechanisms or patient-specific variability, and it was not designed for this purpose.

**Article 3 – Yilgor C, Sogunmez N, Boissiere L, et al. Global Alignment and Proportion (GAP) Score: Development and Validation of a New Method of Analyzing Spinopelvic Alignment to Predict Mechanical Complications After Adult Spinal Deformity Surgery. J Bone Joint Surg Am. 2017;99(19):1661-1672.z.01594^5^**

### Clinical Rationale

Despite major advancements in surgical correction of adult spinal deformity (ASD), mechanical complications such as proximal junctional kyphosis (PJK) and pseudarthrosis are prevalent. These complications contribute to morbidity, reduce patient satisfaction, and increase healthcare costs.

Historically, surgical planning and postoperative assessment have relied on classical sagittal parameters such as pelvic incidence minus lumbar lordosis (PI–LL) mismatch, C7 sagittal vertical axis (SVA), and pelvic tilt (PT). While these traditional alignment parameters offer a foundational framework for evaluating sagittal alignment, they exhibit important limitations and are not precise. These targets do not consistently correlate with reduced mechanical complications. By focusing on isolated regions or posture-dependent measurements, traditional parameters also overlook the global relationship between the spine and pelvis, which function as a unified biomechanical system. Finally, the use of fixed cutoffs imposes artificial categorical boundaries on what is inherently a continuum, potentially missing subtle but clinically relevant degrees of malalignment.

To address these challenges, the authors introduced the Global Alignment and Proportion (GAP) Score, a novel quantitative model of spinopelvic alignment based on the principle of proportionality to PI. Rather than fixed numeric targets, this framework incorporates individualized, morphology-based alignment goals aiming to enhance predictive accuracy of mechanical complications (Figure 1).

### Study Summary

This retrospective multicenter study (conducted across 6 centers in Europe) included 222 adult patients who underwent at least 4 levels of posterior instrumented spinal fusion with a minimum of 2-year follow-up. Inclusion in the European database required the presence of at least 1 of the following radiographic deformity features: coronal Cobb angle ≥20°, SVA ≥5 cm, PT ≥25°, or thoracic kyphosis ≥60°. To assess sagittal alignment in a morphology-based and individualized manner, the authors developed the GAP Score (Table) by integrating 4 radiographic parameters—each specific to any particular pelvic incidence (PI)—along with an age adjustment factor. The score accounts for relative pelvic version, relative lumbar lordosis, lordosis distribution index, and relative spinopelvic alignment. Postoperative spinal alignments were scored and patients placed into 1 of 3 groups: proportioned (0-2 points), moderately disproportioned (3-6 points), and severely disproportioned (≥7 points), establishing a risk-based framework for mechanical complications.

Patients classified as “proportioned” experienced a mechanical complication rate of just 6%, compared to 47% in the “moderately disproportioned” group and 95% in the “severely disproportioned” group. Corresponding rates of mechanical revision surgery were 3%, 21%, and 55%, respectively. Additionally, significant differences in health-related quality-of-life outcomes were observed across GAP score categories, with proportioned patients reporting markedly better functional scores.

### Methodological Review

This study has several notable strengths. It introduces an innovative approach by using regional and global sagittal alignments that are based on each patient’s pelvic incidence, moving beyond fixed numerical thresholds. The analysis was conducted on a large, multicenter European cohort, enhancing the external validity of the findings. The study used objective and relevant outcome: mechanical complications, and the score was based on normative alignment, and incorporated a metric of lordosis distribution. The GAP score is built on clear and reproducible parameters that can be reliably assessed using standard preoperative and postoperative imaging, supporting its utility in surgical planning and postoperative evaluation. Additionally, the inclusion of an internal validation cohort within the study lends credibility to the scoring system’s predictive capacity for mechanical complications after ASD surgery.

However, certain limitations should be acknowledged. The retrospective design introduces potential for selection bias and limits causal inference. The study lacked an external validation cohort, raising questions about the applicability of the GAP score to broader or more diverse populations, including those with different surgical practices or patient demographics. Furthermore, subsequent external validation studies have shown slightly worse performance of the GAP score, though this is common when comparing internal and external validation studies. In addition, the study used a classifier, as opposed to a probability model, there is potential for collinearity of the alignment metrics, and incorporation bias when including Relative Spinopelvic Alignment. Finally, some of the postural measures cannot be assessed intraoperatively, and therefore have limited use as a surgical alignment target.

### Quality of Evidence: Moderate

Internal validity supported by large sample size and structured scoring. Predictive power may be limited in elderly or osteoporotic populations.

### Recommendation for Integrating Into Clinical Practice

The GAP Score provides a structured, pelvic incidence-based framework for evaluating spinopelvic alignment and stratifying the risk of mechanical complications following ASD surgery.

### GRADE Recommendation: Conditional

**We conditionally recommend** incorporating the GAP Score into postoperative assessment for spinal fusion surgery, recognizing its utility in pelvic incidence-based alignment evaluation. Although the scoring system is relatively straightforward to apply using standard postoperative standing radiographs, it is not designed for intraoperative use, as several required parameters cannot be measured in the prone position. Thus, the GAP Score is best used in the preoperative planning phase and for postoperative alignment assessment, where it can help surgeons contextualize alignment goals and estimate complication risk based on patient morphology. While promising in its ability to predict mechanical complications, it is not designed as a stand-alone prediction tool for mechanical complications. It is best used alongside clinical judgment and other individualized patient assessments to guide surgical planning.

**Article 4 – Hills J, Mundis GM, Klineberg EO, et al. The T4-L1-Hip Axis: Sagittal Spinal Realignment Targets in Long-Construct Adult Spinal Deformity Surgery. J Bone Joint Surg Am. 2024;106:e48(1-10). doi:10.2106/JBJS.23.00372^6^**

### Clinical Rationale

Widely used alignment targets—such as PI–LL mismatch or classification systems—have shown inconsistent reproducibility across ASD cohorts. A key limitation of these frameworks is their reliance on postural parameters (eg, PT or SVA) and categorical thresholds. Moreover, in normal spines, PI–LL mismatch demonstrates wide variability, limiting its utility as a precise surgical target. While a step forward, the GAP Score was limited by its use of postural measures (relative pelvic version) and the imprecise use of categories to classify continuous variables.

To address these shortcomings, prior work introduced the T4–L1–Hip axis as a means of defining normal sagittal alignment based on a cohort of adults without spinal deformity or degeneration. This approach utilizes vertebral-pelvic angles—specifically the L1 pelvic angle (L1PA) and T4 pelvic angle (T4PA)—which are directly modifiable during surgery and more closely linked with PI and global sagittal alignment than traditional Cobb-based measures. The T4–L1PA mismatch provides a continuous metric for aligning the thoracic spine relative to the lumbar spine, offering a practical and anatomically grounded framework for surgical planning (Figure 1). The current study sought to determine whether realignment to normal sagittal alignment based on the T4-L1-Hip axis was associated with reduced risk of mechanical complications following long-construct fusion to the sacrum in adult spinal deformity patients.

### Study Summary

This multicenter retrospective study analyzed 247 patients with adult spinal deformity who underwent long-construct fusion from the upper thoracic spine (T1–T5) to the sacrum or pelvis. The objective was to determine whether realignment to a normal T4–L1–Hip axis was associated with a reduced risk of mechanical complications (PJK, rod fracture, or revision for either) within 2 years. Mechanical complications occurred in 28% of patients. The authors found significant and nonlinear associations between complication risk and deviation from a normal L1PA and T4–L1PA mismatch. The lowest risk of mechanical complication was observed when L1PA was 0.5 × PI – 19° ± 2°, and the T4–L1PA mismatch was between −3° to +1°. Deviations outside these ranges (overcorrection or undercorrection) were independently associated with increased risk of mechanical failure.

Postoperative changes in T4PA were nearly perfectly correlated with changes in C2 pelvic angle (C2PA) (r^2^ = 0.96), and higher postoperative C2PA was significantly associated with worse 1-year patient-reported outcomes across all SRS domains and the Oswestry Disability Index. These findings support the use of T4PA and L1PA as directly modifiable intraoperative alignment targets that influence both mechanical durability and functional outcomes.

### Methodological Review

Strengths include a relatively large sample of patients undergoing long-construct fusion, minimizing confounding from UIV variability. Outcomes were objectively defined and confirmed by 2 independent spine surgeons. The statistical analysis was robust, using multivariable nonlinear regression with continuous variables and a probability-based modeling approach—avoiding arbitrary thresholds and improving clinical relevance. Importantly, the alignment metrics (L1PA and T4–L1PA mismatch) are geometrically tied to sagittal balance and are directly measurable and modifiable intraoperatively.

Limitations include its observational design, with potential for unmeasured confounding, such as preoperative alignment (especially cervicothoracic), and technical surgical factors (performance bias). Patient-specific factors, including preoperative alignment, bone quality, and surgical goals, may also affect surgical and alignment planning, and these alignment targets must be considered as a part of the entire evaluation. Subsequent analyses have found that the T4-L1PA relationship varies with pelvic incidence, with lower PI having a larger T4PA while higher PI have a lower T4PI relative to L1PA. Mechanical failure definitions, though standardized, remain susceptible to misclassification. Importantly, the findings apply only to long construct fusions, thus limiting the clinical utility if not instrumented from the upper thoracic spine to the sacrum. Lastly, external validation is needed.

### Quality of Evidence: Moderate

The primary strength is use of a nonlinear probability model using metrics that can be measured intraoperatively, and main limitation is clinical utility limited to long-construct fusions.

### Recommendation for Integrating Into Clinical Practice

Realignment goals based on the T4-L1-Hip axis represent alignment parameters that are directly modifiable and measurable intraoperatively and were associated with a reduced risk of mechanical complications and improved functional outcomes in this study. The probability-based modeling approach used—rather than categorical thresholds—offers a more flexible and potentially more accurate framework for alignment planning compared to classification-based strategies.

### GRADE Recommendation: Conditional

Given the observational nature of the study and need for external validation, **we offer a conditional recommendation** to incorporate L1PA and T4–L1PA mismatch as alignment targets in preoperative planning and intraoperative assessment for long-construct deformity cases. L1PA and T4–L1PA mismatch should be interpreted in the context of patient factors, treatment goals, and risk tolerance. Although optimizing these parameters may reduce the risk of mechanical failure in long fusions to the pelvis, they represent regional alignment targets and do not define segment-specific alignment objectives.

## Discussion

### Limitations of Radiographic-Only Planning

Historically, sagittal realignment targets in ASD surgery were derived from radiographic norms observed in asymptomatic adults.^3,4^ While these parameters—such as PI—LL, PT, and SVA—correlate with disability when grossly abnormal,^4,11^ their direct application to surgical planning lacks specificity. As highlighted by Le Huec et al., spinal alignment is not a fixed geometric concept but a dynamic interplay between the spine, the pelvis, and the lower extremities that adapts to age, pathology, and compensation.^3^

Current planning systems fail to incorporate critical patient-specific risk factors such as age, bone mineral density (BMD), muscle quality, and comorbidities—variables known to influence complication rates and long-term function. Thus, while the conceptual understanding of alignment has matured, clinical implementation remains limited by the oversimplification of complex, individualized factors.

A purely radiographic approach also neglects intrinsic patient factors—most notably, the biological reserve that determines whether a patient can tolerate a given correction.^12,13^ For example, achieving “perfect” alignment goals in a patient not suitable for a major reconstruction may increase the risk of perioperative morbidity. Therefore, the goal should be to “normalize” alignment, while understanding the constraints of what any single patient may tolerate in the operating room.

### A Multimodal Strategy

The 4 reviewed publications together form the historical foundation for sagittal alignment assessment and planning in ASD surgery. Each provides a unique perspective—ranging from classification to proportionality to physiological references and morphological typology. Their collective value lies not only in their individual insights but in how they contribute to a multidimensional understanding of spinal balance, which is essential for modern deformity correction. While no single model currently offers perfect predictive accuracy, each study has useful information for discussion/decision-making:

• **The Roussouly classification grouped patients by sacral slope, with corresponding “shapes” defined by apices of curvature and inflection points,**
• **The SRS-Schwab classification allows for categorization of deformity, though several classification modifiers lack the precision needed for individualized alignment targets,**
• **The GAP score offers pelvic incidence-based targets for regional and global alignment, with the goal of reducing mechanical complications,**
• **The T4-L1-Hip axis described spine shape in a continuous manner, using pelvic-incidence as a fixed measure. Deviations from normal L1PA and T4-L1PA are associated with higher risks of mechanical complication.**

### Clinical Implications and Future Directions

Looking ahead, future alignment frameworks must evolve beyond risk stratification and towards a true decision-support system. This includes guiding the surgical team in balancing competing objectives: achieving alignment without exceeding biological tolerance, correcting deformity while preserving stability, and optimizing durability. A meaningful planning strategy must navigate these factors on an individual basis.

The key takeaway from this review is not to rank alignment models, but to recognize how each has incrementally advanced our understanding of sagittal alignment. Collectively, they mark a shift from descriptive morphology toward more proportional and continuous measures of alignment. Each model offers insights and addresses limitations of the models that preceded it, reflecting an evolving understanding of how spinal shape and global balance influence outcomes in ASD surgery. Rather than applying any single model in isolation, surgeons should understand the strengths and limitations of each framework and integrate this knowledge with patient-specific factors—such as goals, comorbidities, bone quality, and physiologic reserve—when formulating alignment targets and surgical strategies.
